# Retrospective survival in elderly COPD patients receiving pulmonary rehabilitation; a study including maintenance rehabilitation

**DOI:** 10.1186/1756-0500-7-210

**Published:** 2014-04-03

**Authors:** Audhild Hjalmarsen, Tormod Brenn, Marijke Jongsma Risberg, Kathrine Meisler Antonsen, Elisabeth Kristiansen Benum, Ulf Aaseboe

**Affiliations:** 1 Department of Clinical Medicine, University of Tromsoe, Tromsoe N 9038, Norway; 2 Department of Community Medicine, University of Tromsoe, Tromsoe, Norway; 3 Department of Physioterapy, University Hospital of North Norway, Tromsoe, Norway; 4 Department of Pediatry, Central Hospital of Nordland, Bodoe, Norway; 5 Department of Medicine, General Hospital of Helgeland, Sandnessjoen, Sandnessjoen, Norway

**Keywords:** Copd, Elderly, Pulmonary rehabilitation, ltot, Survival

## Abstract

**Background:**

The aim of this study was to examine retrospective survival in elderly chronic obstructive pulmonary disease (COPD) patients receiving three different pulmonary rehabilitation (PR) programs.

**Results:**

193 patients [m / f 92 / 101, mean age 69.2 (standard deviation 8.6)] receiving PR were studied with lifetable and Cox regression analyses. Forced expiratory volume in 1 second (FEV_1_) % pred. was significantly different in the in-patient (n = 72), out-patient (n = 72), and maintenance group (n = 49) [mean 54.5 (21.8), 52.2 (17.7), and 42.9 (15.0), respectively (p = 0.004)]. PR days were 30.3 (20.4), 18.9 (10.4) and 30.0 (20.3), respectively (p < 0.001). Median survival rate was nine years in the in-patient, eight years in the out-patient and seven years in the maintenance group. Hospital stays and days were significantly increased in the maintenance group compared with the other groups (p = 0.003 and 0.010, respectively). The impact of evaluated variables on survival in the three PR groups was significant for age, FEV_1_ as well as the use of long-term oxygen therapy (LTOT) (HR 1.06, for five years, p < 0.001, HR 0.98, p = 0.01, and HR 2.18, p = 0.005, respectively).

**Conclusions:**

The COPD patients in the maintenance group showed a worse survival, but after correction for gender, age and severity of obstruction, the difference was not statistically significant.

## Background

Pulmonary rehabilitation (PR) is a multidisciplinary intervention for the management of chronic obstructive pulmonary disease (COPD) [1-3]. The effect of PR is reduction of dyspnea, increased functional exercise capacity and improved health related quality of life (HRQL) [4-10]. Several previous studies have shown increased survival in patients with COPD receiving PR [11-13]. However, only one prospective randomized, controlled study of PR has evaluated the effect on long-term survival. Ries and colleagues randomly assigned patients with COPD to either an 8-week comprehensive outpatient PR program (57 patient) or to a control group given educational sessions (62 patients). Although 67% of the rehabilitation group versus 56% of the education group were still alive at 6 years, the difference was not statistically significant (p = .3) [14].

Ries and colleagues included an analysis of health care use. Even though the number of hospital days decreased by 2.4 days in the rehabilitation group and increased by 1.3 days for the control group, the difference was not statistically significant (p = .2). Bourbeau and colleagues showed that hospitalisation for COPD exacerbations decreased by 39.8% with intervention that consisted of a comprehensive patient education program compared with a group that received usual care [15]. Self-management education is complementary to exercise training in pulmonary rehabilitation. Two similar studies with before and after design have also demonstrated health care use reductions [16,17].

A systematic review showed that supervised exercise programs after primary PR appear to be more effective than usual care for preserving exercise capacity after six and 12 months. The small number of studies precluded a definitive conclusion as to the impact of postrehabilitation exercise maintenance on longer-term benefits in COPD patients [18].

The aim of this study was to report the retrospective 10 years survival of elderly patients with COPD stage 1 – 4 [19], as measured by forced expiratory volume in one second (FEV_1_) % predicted, receiving three different PR programs, in-patient, out-patient and maintenance program. The role of maintenance pulmonary rehabilitation intervention following initial structured programs is still uncertain [20]. However, we supposed that COPD patients receiving maintenance rehabilitation should have poorer survival because of increased morbidity and more frequent hospitalizations for exacerbation. A secondary aim was to report the use of long-term oxygen therapy (LTOT) because of it’s life-longing effect in chronic respiratory failure [21-23].

## Methods

### Patients

In retrospect we selected 200 patients with the diagnosis of COPD receiving PR at two different centres in Northern Norway (Skibotn Rehabilitation Centre, Skibotn and St Elisabeth Centre, Tromsoe) between 1993 and 2004. These were the first COPD patients receiving PR at these centres. Patients with other pulmonary disease, coronary heart disease, stroke or severe systemic disease were not included. The patients included lived in the same geographical area and were the catchment population of the University Hospital of North Norway. The baseline population was about 200 000. The catchment population of each centre were similar with respect to urban/non-urban area. Data was collected from the hospital patient records. Seven patients moved out of the area and were excluded from the study. 193 patients (92 males and 101 females of mean age 69.2 years) were included (Table 1). 72 patients received in-patient rehabilitation at Skibotn Rehabilitation Centre, 72 patients received out-patient and 49 received maintenance rehabilitation at St Elisabeth Centre. Each study patient was included in one PR group only. The follow-up time started at the beginning of the PR. The patients were censored at start of the program. The date of death was reported by the Statistics Norway directly to the patients electronic hospital record.

**Table 1:** Patient characteristics of the three groups of COPD patients selected according to type of PR program*

	**Total**	**In**-**patient group**	**Out**-**patient group**	**Maintenance group**	***P*-value**^**†**^
	**(n = 193)**	**(n = 72)**	**(n = 72)**	**(n = 49)**	
Age, yrs	69.2 (8.6)	67.5 (8.2)	70.4 (9.1)	69.9 (8.2)	0.11
Sex, male (%)	92 (47.7)	41 (56.9)	32 (44.4)	19 (38.8)	0.11
BMI, kg/m^2^	25.2 (6.1)	25.4 (5.6)	25.4 (5.6)	24.8 (6.9)	0.85
FVC Litres	2.25 (0.81)	2.53 (0.97)	2.23 (0.68)	1.92 (0.55)	< 0.001
FVC % predicted	70.8 (20.1)	74.7 (20.6)	71.5 (19.8)	63.8 (18.3)	0.010
FEV_1_, Litres	1.30 (0.62)	1.50 (0.76)	1.29 (0.51)	1.03 (0.39)	< 0.001
FEV_1_ % predicted	50.7 (19.6)	54.5 (21.8)	52.2 (17.7)	42.9 (15.0)	0.004
FEV_1_/FVC %	56.3 (14.6)	56.5 (14.5)	57.8 (14.3)	53.2 (14.4)	0.167
PaO_2_ kPa (air)**	9.37 (1.33)	9.55 (1.07)	9.36 (1.37)	9.12 (1.61)	0.209
PaCO_2_ kPa (air)**	5.48 (0.93)	5.35 (0.88)	5.49 (0.83)	5.66 (1.12)	0.199
6 min WD, m***	359.6 (135.7)	379.9 (128.8)	367.4 (158.4)	316.4 (104.1)	0.060
PR days	26.1 (18.2)	30.3 (20.4)	18.9 (10.4)	30.0 (20.3)	< 0.001
Hospital stays^††^	0.78 (1.35)	0.35 (0.85)	0.86 (1.46)	1.17 (1.60)	0.003
Hospital days^††^	5.2 (11.1)	2.90 (8.89)	4.78 (8.26)	9.04 (15.69)	0.010
LTOT, %^†††^	37.8	26.4	29.2	67.3	< 0.001
Survival, yrs	7.3 (3.5)	8.1 (3.6)	6.7 (3.1)	7.1 (3.9)	0.042
Mortality, %^††††^	53.9	50.0	47.2	69.4	0.056

*Data are shown in mean (s) with standard deviation in parenthesis. ^†^Testing means or percentages across groups. **Group numbers are 179, 68, 64 and 47, respectively. ***Group numbers are 165, 69, 57 and 39, respectively. ^††^Group numbers are 191, 71, 72 and 48, respectivley. ^†††^Group numbers are 73, 19, 21 and 33, respectively. ^††††^ Group numbers are 104, 36, 34 and 34, respectively.

COPD = chronic obstructive pulmonary disease; s = standard deviation; BMI = body mass index; FVC = forced vital capacity; FEV1 = forced expiratory volume in 1 second; PaO_2_ = partial arterial oxygen pressure (air); PaCO_2_ = partial arterial carbon dioxide pressure (air); 6 minWD = 6 minutes walking distance. PR = pulmonary rehabilitation; LTOT = long-term oxygen therapy.

### Rehabilitation program and treatment

The PR programs were conducted by the hospital. The PR team consisted of pulmonary physician, geriatrician, respiratory nurse, physiotherapist, occupational therapist, specialist in nutrition and social worker. The in-patient program lasted four weeks and included 20 days of comprehensive pulmonary rehabilitation. This program implied mobile patients without need of nursing during daily activities. The out-patient program included ambulant pulmonary rehabilitation two days a week for 8 weeks. The study patients had participated in the in- or out-patient program at least 75% of the time. Both programs included 12 lectures of patient education and two hours of exercise training daily, including endurance and strength training. Ventilatory muscle training was part of the exercise program. Patients with mild and moderate COPD practised high-intensity endurance training, while patients with severe hypoxaemia were trained in low-intensity exercise keeping the SpO_2_ above 85% and the pulse below 130 per minute during activity. Oxygen was supplied when needed. The endurance and strength training programs involved both upper- and lower extremity training on fitness centre equipment including armergometer, ergometercycle and treadmill. The maintenance program always started with an in- or out-patient program and then continued with an one or twice a week follow-up rehabilitation program for as long as the patient needed it or was strong enough to participate. During exacerbation or other causes they stopped temporarily. The maintenance program was therefore of individual length. However, the patients could return to the same group when needed. The maintenance program was as comprehensive as the in- and out-patient programs, but adapted to the very severely diseased COPD patients.

The maintenance program included education, psychosocial activation, endurance and strenght training. COPD patients with FEV_1_ < 35 percent of predicted and those using LTOT or ambulatory oxygen therapy were included in the maintenance group. The majority of these patients had then stopped smoking.

LTOT was prescribed according to the international presciption guidelines [21-23]. The standards for LTOT were PaO_2_ ≤ 7.3 kPa while breathing air, with measures taken at least three times during stable disease. If coexisting polycythemia or cor pulmonale, LTOT started up to a resting PaO_2_ of 8 kPa. Two patients in the maintenance group received oxygen therapy on even higher levels of PaO_2_, due to severe dyspnea on effort.

Additional treatments included beta_2_-agonists, inhaled corticosteroids and/or sustained release methylxanthines. Diuretics, antibiotics and oral corticosteroids were used as clinically indicated.

Permission for collecting of data from the hospital records was given by the authorities at the University Hospital of North Norway as a quality control to improve our treatment practice. The access to the database is not freely available. Permission to use it is given by the Department of Clinical Medicine, University of Tromso. The study was also approved by the Regional Committee of Research Ethics of North Norway, REK nord – Region Nordland, Troms and Finnmark, located at the University of Tromso.

### Measurements

Baseline registration included history and clinical examination, electrocardiography (ECG), chest x-rays, spirometry, arterial blood gas analysis, 6 minutes walking distance (6-minWD), and a collection of blood tests including hemoglobin and C-reactive protein (CRP). European reference values for spirometry were used. The smoking status was not described detailed enough in the patients records to calculate the package years. The study patients were mainly ex-smokers, but several were still-smokers at start of the PR. Because of the long observation time the smoking status became inestimable. Post-bronchodilator FEV_1_/FVC < 0.70 was used to confirm the presence of airflow limitation and thus of COPD. Based on post-bronchodilator FEV_1_ we used the Global Initiative for Chronic Obstructive Lung Diseases (GOLD) classification to assess the degree of airflow limitation severity in COPD. GOLD stage 1, mild degree, was defined by a FEV_1_ ≥ 80% of predicted, stage 2, moderate degree, by FEV_1_ < 80 and ≥ 50% of predicted, stage 3, severe degree, by FEV_1_ < 50 and ≥ 30% of predicted and stage 4, very severe degree, by FEV_1_ < 30% of predicted [19].

### Statistical analysis

Patients were allocated into three groups according to type of PR program (in-patient, out-patient and maintenance group). Numeric data were expressed as mean (s) with standard deviation in parenthesis. One-way analysis of variance was employed to compare means in different groups. A chisquare - test was used for the categorial variable sex. Survival curves were derived by the Kaplan-Meier method. Cox’s proportional hazards regression model [24] was used to assess the impact on survival of the different explanatory variables including, age, gender, PaO_2_, PaCO_2_, FEV_1_, FVC, FEV_1_/FVC, body mass index (BMI) and COPD stage. The importance of the different variables was examined both by univariate and multivariate analysis to find the combination of factors that could predict early mortality. When comparing the three types of PR program, the maintenance program served as reference (HR of 1.0). The COPD stage 1 served as reference when comparing the four stages. Male served as reference when comparing the two sexes. A p-value of < 0.05 was considered statistically significant. The computer program SPSS was used for statistical analysis (SPSS, Chicago, IL, USA).

## Results

Seventy-two patients with mean FEV_1_ 54.5% pred., 72 with mean FEV_1_ 52.2% pred., and 49 with mean FEV_1_ 42.9% pred. receiving three different rehabilitation programs were allocated to three groups; the in-patient, the out-patient and the maintenance group, respectively (Table 1). Two of the patients included in the maintenance group showed a FEV_1_ % predicted of 73.6 and 51.8, respectively, and received ambulatory oxygen therapy. Three LTOT patients with predominating emphysema were also included in this group despite high values of FEV_1_ % predicted of 84, 77.8 and 72.2, because they suffered from severe chronic hypoxaemia with resting PaO_2_ levels of 6.41, 6.51 and 5.8 kPa, respectively. According to the FEV_1_ % pred., 18 patients (9.3%) were allocated to COPD stage 1, 74 (38.3) to stage 2, 79 (40.9%) to stage 3, and 22 (11.4%) to stage 4 (not in tables). Mean survival time and mortality rate are reported in Table 1. At the end of the observation period totally 90 patients were still alive, 36 in the in-patient, 39 in the out-patient and 15 in the maintenance group.

**Figure 1:**
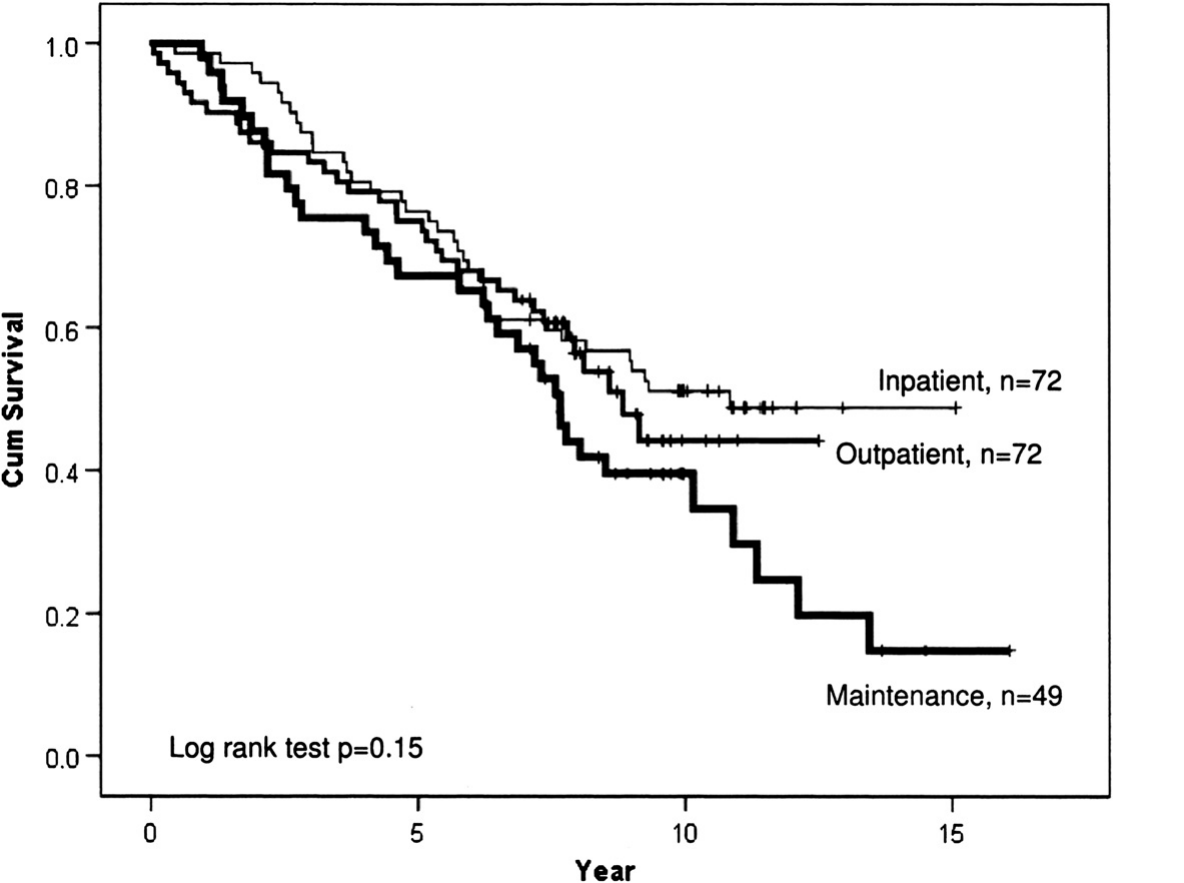
Cumulative survival of COPD patients receiving different pulmonary rehabilitation program, in-patient, out-patient or maintenance.

**Figure 2:**
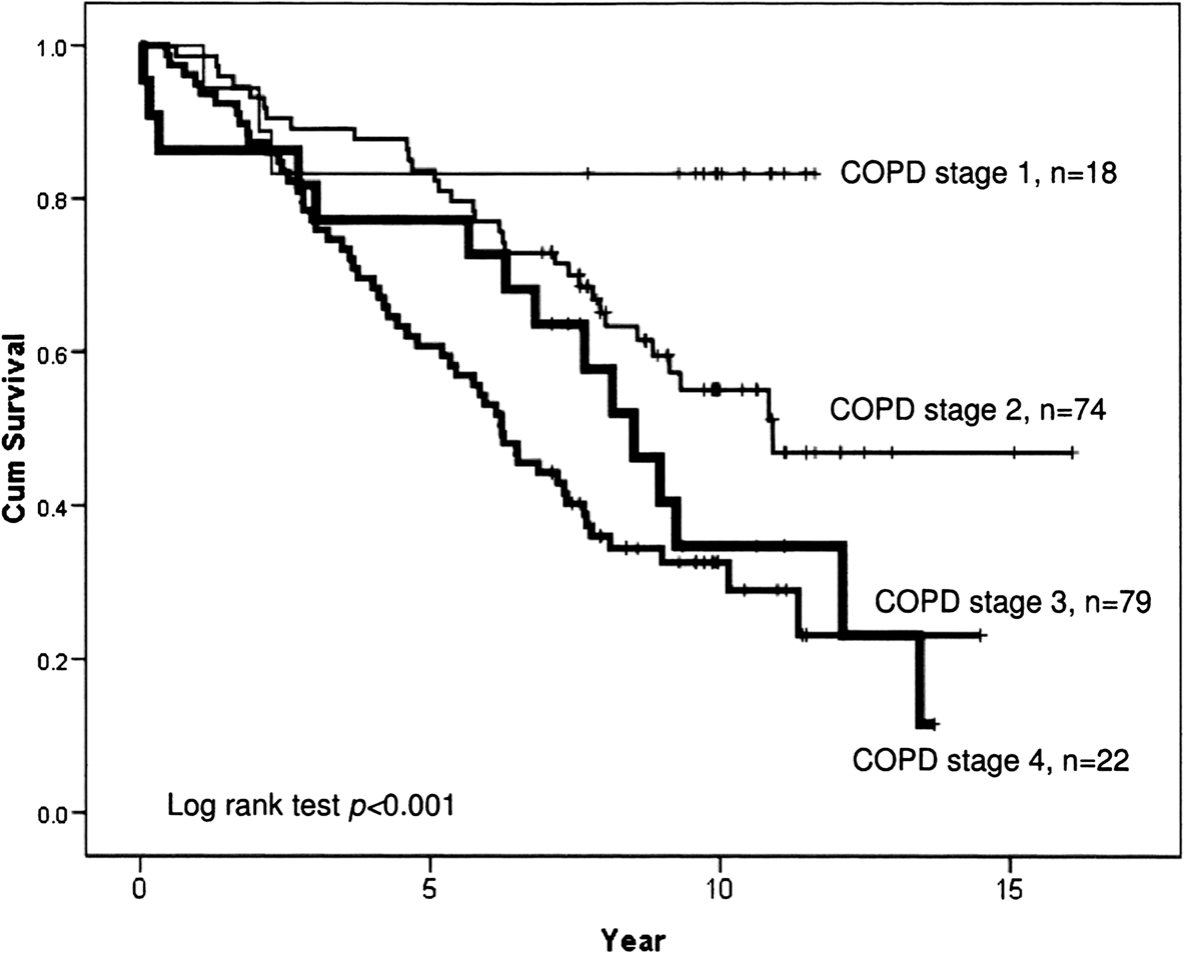
Cumulative survival of COPD patients in the four stages.

Figure 1 show survival, and as seen the five and ten year survival rates were 75 and 55% in the in-patient, 70 and 45% in the out-patient, and 65 and 40% in the maintenance group, respectively. Median survival rate was nine years in the in-patient, eight years in the out-patient and seven years in the maintenance group. Figure 2 show that five and ten year survival rates were both 85% in stage 1, 85 and 55% in stage 2, 55 and 30% in stage 3, and 75% and 35% in stage 4, respectively. Median survival rate was eight years in COPD stage 4, six years in stage 3, and >10 years in stage 1 and 2.

The impact of the evaluated variables on survival in the three PR groups was significant for age, FEV_1_ % predicted as well as the use of LTOT at the registration date (HR 1.06, for five years, p < 0.001, HR 0.98, p = 0.01, and HR 2.18, p = 0.005, respectively) (Table 2). The impact of the evaluated variables on survival in the four COPD stages was significant for age, LTOT and COPD stage 3 (HR 1.05, p < 0.001, HR 2.33, p < 0.001, and HR 3.41, p = 0.04, respectively) (Table 3). The impact of 6-min WD on survival was significant for COPD stage 3 and 4 (HR 1.00, for five years, p < 0.001) (not in tables).

**Table 2:** **Impact of evaluated variables on survival of COPD patients in the three pulmonary rehabilitation programs according to Cox regression analysis** (**n** = **193**)

	**HR(95% CI)**	***P*-value**
Age	1.06* (1.03 - 1.09)	< 0.001
Sex	0.88** (0.56 - 1.38)	0.57
BMI (kg/m^2^)	0.99 (0.95 - 1.03)	0.59
FEV_1_ % predicted	0.98*** (0.97 - 1.00)	0.01
PaO_2_ kPa (air)	0.88 (0.73 - 1.07)	0.21
PaCO_2_ kPa (air)	0.77 (0.59 - 1.01)	0.06
LTOT	2.18 (1.33 - 3.56)	0.005
PR program		
In-patient	0.89 (0.53 - 1.50)	0.65
Out-patient	0.75 (0.44 - 1.28)	0.30
Maintenance	1.0 (ref.)	

The variables were mutually adjusted for all other variables in the table.

HR = hazard ratio; CI = confidence interval; LTOT = long term oxygen therapy;

Ref. = reference; for other abbreviations see Table 1.

*5 years; **Male served as reference; ***10%.

**Table 3:** **Impact of evaluated variables on survival of COPD patients at the four stages according to Cox regression analysis** (**n** = **193**)

	**HR(95% CI)**	***P*-value**
Age	1.05* (1.02 - 1.08)	< 0.001
Sex	0.68** (0.44 - 1.05)	0.08
BMI (kg/m^2^)	0.98 (0.94 - 1.02)	0.32
PaO_2_ kPa (air)	0.85 (0.70 - 1.02)	0.09
PaCO_2_ kPa (air)	0.84 ( 0.64 - 1.09)	0.19
LTOT	2.33 (1.44 - 3.77)	< 0.001
COPD GOLD stage		
1 (FEV_1_ ≥ 80% predicted)	1.0 (ref.)	
2 (FEV_1_ 50–80% predicted)	1.90 (0.57 - 6.35)	0.30
3 (FEV_1_ 30–50% predicted)	3.41 (1.30 - 11.25)	0.04
4 (FEV_1_ < 30% predicted)	1.84 (0.47 - 7.14)	0.38

Abbreviations see Tables 1 and 3.

The variables were mutually adjusted for all other variables in the table.

*5 years; **Male served as reference.

Mean days of PR in the in-patient, out-patient and maintenance group were 30.3 (20.4), 18.9 (10.4) and 30.0 (20.3), respectively (p < 0.001). Mean hospital stays the year before the start of PR were 0.35 (0.85) (n = 71), 0.86 (1.46) (n = 72), and 1.17 (1.60) (n = 48), respectively (p = 0.003), and mean days in hospital were 2.90 (8.89) (n = 71), 4.78 (8.26) (n = 72), and 9.04 (15.69) (n = 48), respectively (p = 0.010) (Table 1).

Patients using LTOT were recorded from 1993 until the end of the observation period in 2012. Totally 73 patients used or had been using LTOT, 19 in the in-patient group, 21 in the out-patient group and 33 in the maintenance group (Table 1). According to COPD stage as measured by FEV_1_ at the start of the rehabilitation period, one patient in stage 1, 25 in stage 2, 32 in stage 3 and 16 in stage 4 were on LTOT (not in tables).

In the maintenance group 30.4% started with an in-patient program and 69.6% with an out-patient program. Mean survival was 8.1 years (standard deviation 4.1) and 6.4 (3.5), respectively (p = 0.070) (not in tables).

## Discussion

This retrospective study present the 10 year survival of elderly COPD patients receiving PR according to three modalities, four weeks in-patient, eight weeks out-patient and maintenance rehabilitation on a weekly basis following an in- or out-patient program. We found significant differences in severity of COPD between groups. However, the statistical analyses showed several risk factors for increased mortality like very severely decreased FEV_1_, the need of LTOT and significantly increased hospitalizations for exacerbation. The maintenance group had worse survival, but after correction for gender, age and severity of obstruction, the difference was not statistically significant.

When evaluating the COPD stages, the five and ten years survival was 55 and 30 percent in stage three and 75 and 35 percent in stage four. In severe COPD not only FEV_1_, but also BMI, package years, smoking status, exacerbations and 6-minWD predict survival [25,26]. In this study COPD patients in stage three showed an insignificantly increased mortality compared to patients in stage four. This may be explained by the group size difference. In accordance with previous knowledge, we found that older age, decreased pulmonary function and LTOT were significant predictors of survival in all groups [21-23,25,26].

There was a significant difference in both stays and days in hospital when comparing the PR groups the year before rehabilitation. However, despite fewer stays and days in the in-patient group compared to the out-patient group, the number of rehabilitation days were significantly higher, mean 30.3 versus 18.9, respectively. Patients in the out-patient group had less PR days and more hospital days. So, it seems that the savings on PR days are offset by increased expenses on hospital days. The in-patient group received the same number of rehabilitation days as the maintenance group. Several patients in the in-patient group had more than one rehabilitation, whereas the maintenance group attended an out-patient follow-up program on a weekly basis over time. Because of more hospital days in the maintenance group it is still uncertain whether it is more cost-effective with maintenance rehabilitation in an out-patient setting than in-patient rehabilitation. The maintenance group consisted of patients commonly in need of home nursing or caregiving relatives, while the in-patient group had to be independent as this centre was situated in an non-urban area far from the hospital. This study also showed that it was possible to treat the very severely diseased COPD patients favourably in an out-patient setting for a longer period of time.

The number of patients are limited. The reason is that since 2005 we offered maintenance rehabilitation to COPD patients in COPD stage three as well. Other variables, which are known to be predictors of survival, like package years and smoking status were not recorded adequately enough in the patients records over time. All the study patients had a smoking history. The criteria to receive PR was to have stopped smoking before or confirm to stop smoking when entering the PR program. However, the COPD patients receiving PR may understate the number of cigarettes and relapse is common.

Because the MRC dyspnea scale was not available, we did not use the BMI, airflow obstruction, dyspnea, and exercise capacity (BODE) index. The BODE study [25] showed that 6-min WD is a strong predictor of survival. This study also showed that 6-min WD was a strong predictor of survival in severe COPD. BMI, however, was not found to be a predictor of survival in our study.

As we staged the patients according to the FEV_1_ percent predicted at the start of the PR program, LTOT patients, however, were recorded at the end of the study. Several of the patients had become worse over time. LTOT patients were therefore present in all groups. As COPD patients on LTOT have poorer survival, these patients contributed to the increased mortality in COPD stage two to three.

We did not find an overall gender effect on survival in this study. Though about half of the patients were men, in the spirometric stage four as well, the maintenance group consisted of more women than men. Previous studies have shown better FEV_1_ values in women than in men using LTOT [23]. Although the impact of the evaluated variables was significant for age and FEV_1_ percent predicted, the impact on survival was also very strong for those using LTOT. This is in agreement with previous knowledge [21-23].

We found that COPD stage one and two had little impact on survival. However, after ten years of observation we found increased mortality also in stage two. Many of the patients had then developed a very severe COPD as 25 of these patients had started with LTOT. The reason for the staging according to FEV_1_ percent predicted only was that the patients records at the start of PR did not refer exactly if and when the patient had started with LTOT. Though the study population represents mainly ex-smokers, there were several still-smokers too. Therefore 32 of the PR patients initially staged as severely diseased (GOLD stage 3), later changed to stage four when they became LTOT users.

In the maintenance group we found an insignificantly increased survival (p = .07) in the patients starting with an in-patient program. However, the in-patient group had significantly decreased hospitalisations the year before start, indicating that they were more stable patients with less exacerbation.

The maintenance PR was offered to COPD patients with very severe disease. The goal was to preserve the exercise capacity, prevent hospitalizations and reduce mortality. The format and value of a maintenance program after completing a PR program is still not well defined [20]. In this retrospective study we had no representative controls, because the non-participating COPD patients suffered from considerable comorbidity or were too disabled with an even poorer prognosis.

Prospective randomized studies on survival effect of maintenance rehabilitation in moderate- to severely diseased COPD patients are difficult to accomplish because of the long follow-up. Therefore future studies should focus on reduction of risk factors as an indirect measure of survival effect.

## Conclusions

The COPD patients in the maintenance group showed a worse survival, but after correction for gender, age and severity of obstruction, the difference was not statistically significant. This ten years survival study showed that very severely diseased COPD patients can be favourably treated with a long-term extensive rehabilitation program in an out-patient setting.

## Competing interests

This manuscript has neither financial nor non-financial competing interests.

## Authors’ contributions

AH was pulmonary physician and supervisor of the pulmonary rehabilitation programs, conceived and designed the study, instructed during the data recording and preliminar studies as well as drafting and writing the manuscript, TB performed the statistical analysis, MJR designed and conducted the ambulant exercise training programs, KMA and EKB recorded the data and performed the preliminar studies, UA was the head of the department and participated in coordination and helped to draft the manuscript. All authors read and approved the final manuscript.
